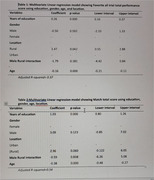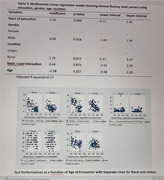# Deployment of Tablet‐based Cognitive Assessment Tool (TabCAT) for detecting cognitive impairment in elderly Primary Health Care Population in Southeast Nigeria

**DOI:** 10.1002/alz.087397

**Published:** 2025-01-09

**Authors:** CHUKWUANUGO OGBUAGU, Richard Uwakwe, James G Kahn, Ekenechukwu OGBUAGU, Obiageli Emelumadu, Uzoma Okereke, Irene Okeke, Chigbo Chisom, Shireen Javandel, Katherine L Possin, Bruce L. Miller, Victor Valcour, Isabel Elaine Allen, Collette A Goode

**Affiliations:** ^1^ Global Brain Health Institute, University of California, San Francisco, CA USA; ^2^ Nnamdi Azikiwe University Teaching Hospital (NAUTH), Nnewi Nigeria; ^3^ University of California San Francisco, San Francisco, CA USA; ^4^ School of Public Health, University of Port Harcourt, Port Harcourt, Rivers Nigeria; ^5^ University of California, San Francisco, San Francisco, CA USA; ^6^ Global Brain Health Institute, University of California, San Francisco, San Francisco, CA USA; ^7^ Memory and Aging Center, Weill Institute for Neurosciences, University of California, San Francisco, San Francisco, CA USA; ^8^ Weill Institute for Neurosciences and Memory and Aging Center, Department of Neurology, University of California, San Francisco, CA USA; ^9^ Global Brain Health Institute (GBHI), University of California San Francisco, San Francisco, CA USA; ^10^ Department of Epidemiology and Biostatistics, University of California, San Francisco, San Francisco, CA USA; ^11^ Memory and Aging Center, UCSF Weill Institute for Neurosciences, University of California, San Francisco, San Francisco, CA USA

## Abstract

**Background:**

Cognitive assessment should be actively incorporated into the clinical evaluation of patients in Primary Health Care (PHC) settings. This is because of the imminent demand with the changing demographics especially in low‐ and middle‐income countries (LMICs) concerning dementia and Alzheimer’s disease.

**Method:**

A cross‐sectional mixed‐method descriptive study was conducted to evaluate the useability and performance of a tablet‐based cognitive assessment tool (TabCAT) for use in geriatric primary healthcare settings in southeast Nigeria.

**Result:**

In our study involving 207 participants, with an average age of 64.69 years, educational levels varied, with 51.8% having primary education, 41.2% secondary, and 6.6% tertiary. The research found that education had a significant positive impact on associative memory, processing speed, executive function, language skills, and visuospatial skills (p<0.001). Rural residents exhibited higher scores in associative memory, processing speed, executive function, language, and visuospatial skills (p<0.000). However, gender and location did not show significant contributions to cognitive performance across the examined domains.

**Conclusion:**

There is an urgent need to deploy and routinely conduct cognitive assessments in primary healthcare clinics for elderly patients for early detection of cognitive changes to improve healthy living.